# Transcriptomic and Epigenetic Reprogramming in *Solanum tuberosum* L. Micropropagation: A Systematic Review of Elicitor-Driven Acclimatization and Scalable Biofactories

**DOI:** 10.3390/ijms27209288

**Published:** 2026-10-19

**Authors:** Guillermo Alexander Jácome Sarchi, Stalin Aldair De La Cruz Sarchi, Jorge Ivan Mina Ortega, Francisca Hernández, Rafael Todos Santos Martínez

**Affiliations:** 1 Grupo de Investigación Agricultura Sostenible (GIAS), Carrera de Agropecuaria, Facultad de Industrias Agropecuarias y Ciencias Ambientales, Universidad Politécnica Estatal del Carchi, Tulcán 040102, Ecuador; 2 Carrera de Alimentos, Facultad de Industrias Agropecuarias y Ciencias Ambientales, Universidad Politécnica Estatal Del Carchi, Tulcán 040102, Ecuador; stalin.delacruz@upec.edu.ec (S.A.D.L.C.S.); jorge.mina@upec.edu.ec (J.I.M.O.); 3 Grupo de Investigación en Fruticultura y Técnicas de Producción, Instituto de Investigación e Innovación Agroalimentaria y Agroambiental (CIAGRO-UMH), Universidad Miguel Hernández, Carretera de Beniel, Km 3.2, 03312 Orihuela, Spain; francisca.hernandez@umh.es

**Keywords:** *Solanum tuberosum*, micropropagation, epigenetic memory, DNA methylation, transcriptomics, plant elicitors, ex vitro acclimatization, abiotic stress

## Abstract

*Solanum* *tuberosum* L. relies heavily on in vitro propagation for pathogen-free seed tuber production; however, the transition to ex vitro environments causes severe plantlet mortality due to transplant shock. Exogenous elicitors have emerged as pivotal preconditioning tools, but their underlying molecular mechanisms remain fragmented. This systematic review aims to elucidate the translational architecture of elicitor-driven preconditioning in *Solanum tuberosum*, systematically organized across a tripartite continuum—(1) epigenetic chromatin plasticity, (2) transcriptomic defense reprogramming, and (3) scalable biofactory platforms (temporary immersion bioreactors and sensor-actuated aeroponics)—required to validate ex vitro physiological competence. Adhering to PRISMA 2020 guidelines for systematic reviews, we conducted a structured search across Scopus, Web of Science, and ScienceDirect (January 2010 to August 2026). Inclusion criteria targeted original research evaluating transcriptomic or epigenetic responses to in vitro elicitation in *S. tuberosum*. Following quality appraisal, data from 58 high-quality studies were synthesized using a critical narrative approach. The evidence is clearly stratified into our three core layers. (1) Transcriptomic Reprogramming: Exogenous elicitors systematically reactivate dormant defense networks prior to ex vitro transfer, converging strictly on salicylic acid (SA), jasmonic acid (JA), and ethylene (ET) signaling pathways alongside reactive oxygen species (ROS) detoxification. (2) Epigenetic Plasticity: Concurrently, morphological adaptation is heavily modulated by reversible chromatin remodeling; specifically, transient DNA hypomethylation and histone acetylation ensure in vitro cellular totipotency and stress memory. (3) Translational Scalability: Automated bioreactors and aeroponic platforms provide the essential physical microenvironment to validate these molecular outcomes and reverse vitrotype disorders at commercial scales. Elicitors drive an integrated tripartite preconditioning continuum that mitigates transplant shock and enables scalable hardening. We delineate a comprehensive translational framework to the transition from empirical micropropagation to targeted, genomics-informed biofactory platforms.

## 1. Introduction

The in vitro propagation of potato (*Solanum tuberosum* L.) is a strategic pillar in global agricultural biotechnology, essential for the continuous regeneration, pathogen sanitation, and mass multiplication of elite germplasm to ensure global food security [1,2]. Despite its critical role, ensuring optimal plantlet quality and successfully navigating the ex vitro acclimatization transition remain formidable bottlenecks [3]. The artificial in vitro environment—characterized by near-saturating relative humidity, low photosynthetic photon flux density, restricted gaseous exchange, and continuous exogenous sucrose supply—induces profound morpho-physiological abnormalities collectively designated as the “vitrotype” [4,5]. This disorder is characterized by six discrete pathological hallmarks: (i) non-functional stomatal apparatuses failing to close in response to darkness or desiccation; (ii) incomplete epicuticular wax deposition and rudimentary cuticles leading to excessive cuticular transpiration; (iii) reduced leaf mesophyll differentiation with poorly organized palisade parenchyma and enlarged intercellular air spaces; (iv) defective, poorly lignified xylem vessels restricting root-to-shoot hydraulic conductivity; (v) suppressed photosynthetic competence caused by sucrose-mediated feedback repression of photosynthetic enzymes; (vi) suppressed baseline immune perception resulting from the sterile, perturbation-free vessel microenvironment [4,5,6]. Consequently, plantlets transferred directly to ex vitro greenhouse conditions suffer catastrophic desiccation, photoinhibition, and acute transplant shock, resulting in severe mortality rates unless targeted physiological preconditioning is deployed [6].

To mitigate transplant shock and enhance phenotypic robustness, recent biotechnological interventions have increasingly focused on the targeted application of exogenous elicitors to induce physiological priming. Within the framework of plant tissue culture, elicitors are operationally defined as biological, chemical, or physical agents that stimulate cellular defense cascades and secondary metabolite biosynthesis upon perception [7]. These stimuli establish physiological priming—the sensitized state enabling plant tissues to mount accelerated and more robust defense responses upon subsequent stress exposure [7,8]—through deliberate in vitro preconditioning, which refers to the controlled manipulation of environmental, chemical, or nutritional regimes to impart physiological vigor prior to ex vitro transfer. Elicitation strategies deployed in micropropagation encompass three primary categories: (i) physical elicitors, including tailored light-emitting diode (LED) spectral regimes and controlled osmotic shifts; (ii) chemical elicitors, spanning foundational phytohormones (salicylic acid, jasmonic acid, and methyl jasmonate), inorganic salts, and bioactive polymers such as chitosan and functionalized nanoparticles; (iii) biological elicitors, comprising plant-growth-promoting rhizobacteria (PGPR, notably *Azospirillum* consortia) and beneficial endophytes [7,8]. Mechanistically, these elicitation regimes operate by triggering membrane-bound receptor-like kinases, transient cytosolic calcium influxes, and enzymatic bursts, which collectively orchestrate systemic resistance [9,10]. During standard in vitro micropropagation, the sterile and highly controlled environment inherently suppresses natural defense networks, contributing to the immunologically vulnerable “vitrotype”. The strategic application of exogenous elicitors is specifically required to artificially reactivate these dormant salicylic acid (SA), jasmonic acid (JA), and ethylene (ET) pathways, alongside ROS detoxification, before ex vitro transfer. This targeted transcriptomic and epigenetic preconditioning effectively pre-arms the plantlets, equipping them with the physiological competence necessary to survive the acute stress of transplant shock [8,9]. Beyond their defensive roles, specific elicitors possess the capacity to simultaneously enhance in vitro morphogenic traits, including shoot multiplication, elongation, and root development [10,11].

Concurrently, *S. tuberosum* micropropagation necessitates profound epigenetic plasticity primarily driven by reversible DNA hypomethylation and dynamic histone H3/H4 acetylation to maintain cellular totipotency and orchestrate stress memory under artificial conditions. Crucially, exogenous elicitors actively modulate this epigenetic landscape, unpacking the chromatin to prime the transcriptomic machinery. This integrated transcriptomic and epigenetic reprogramming allows the rapid transcription of defense-related genes, establishing the essential biological foundation for subsequent ex vitro survival [12,13]. However, a critical epistemological gap persists in the literature. While our recent systematic review in *Horticulturae* [14] synthesized media formulation, light quality, and bio-priming strategies for potato microplants, the underlying cellular reprogramming cascades remain uncoupled. Although high-throughput transcriptomics under single stress regimes have advanced significantly in recent years [8,9], current primary research remains highly fragmented into isolated domains: defense signaling [11], applied micropropagation [1,10], and basic in vitro epigenetic plasticity [12,13]. Consequently, there remains a critical lack of studies linking in vitro epigenetic remodeling (e.g., DNA methylation and histone marks) with transcriptomic defense networks during the transitional ex vitro hardening phase in *S. tuberosum*. A systematic synthesis of the literature is urgently required to map this physiological crosstalk.

In order to address this gap, this systematic review adheres to the PRISMA 2020 guidelines [15] to establish an integrated translational framework evaluating elicitor preconditioning in *S. tuberosum*. We synthesize how nuclear epigenetic remodeling and transcriptomic reprogramming translate into macroscopic vitrotype reversal and critically evaluate how scalable culture platforms—specifically automated bioreactors and IoT-monitored aeroponics—provide the necessary microenvironmental control to deploy preconditioned plantlets at commercial scales.

## 2. Materials and Methods

This systematic review was conducted in strict accordance with the updated Preferred Reporting Items for Systematic Reviews and Meta-Analyses (PRISMA 2020) guidelines [15], ensuring transparency and reproducibility in the selection of scientific literature. A PRISMA 2020 checklist is provided as Supplementary Materials.

### 2.1. Search Strategy

A comprehensive and structured literature search was conducted on 26 August 2026, across three leading electronic databases: Scopus, Web of Science (Core Collection), and ScienceDirect. To ensure procedural feasibility and rigorous screening within an intensive timeline, the evaluation workflow was executed by a dedicated multi-investigator team utilizing automated reference management pipelines and parallel double-blind screening protocols. Furthermore, to capture newly released literature immediately prior to manuscript submission, a final update query was executed on 31 August 2026. The search window encompassed publications from January 2010 to August 2026 to systematically capture contemporary advancements in molecular biology and plant tissue culture.

To ensure reproducibility, search strings were meticulously constructed using Boolean operators (AND, OR) to intersect four core conceptual domains: the target species, the propagation environment, the applied interventions, and the molecular focus. The principal syntax utilized for the Scopus and Web of Science databases was configured as follows: (“*Solanum tuberosum*” OR “potato”) AND (“in vitro” OR “micropropagation” OR “acclimatization” OR “ex vitro”) AND (“elicitor*” OR “nanoparticle*” OR “PGPR” OR “priming”) AND (“transcriptom*” OR “epigenetic*” OR “DNA methylation” OR “histone acetylation” OR “gene expression”). Analogous syntactic adaptations were deployed for ScienceDirect to accommodate platform-specific Boolean limitations (maximum eight connectors), utilizing a condensed string focusing on the primary identifiers: “*Solanum tuberosum*” AND “in vitro” AND (elicitor OR nanoparticle OR PGPR) AND (transcriptomic OR epigenetic).

### 2.2. Eligibility Criteria

To maintain a rigorous focus on the physiological, molecular, and technological foundations of *S. tuberosum* preconditioning and acclimatization, specific eligibility thresholds were established prior to study selection.

Inclusion Criteria: (i) Original, peer-reviewed research articles published in indexed journals providing primary empirical datasets; (ii) investigations providing empirical molecular, epigenetic, or biochemical evidence (such as RNA-seq, RT-qPCR, chromatin profiling, proteomic cascades, or antioxidant enzyme dynamics) responding to in vitro stress or exogenous elicitor application; (iii) studies evaluating physiological competence, hormonal regulation, and morphogenic adaptation during the transition from in vitro culture to ex vitro acclimatization; (iv) research detailing scalable technological platforms (specifically temporary immersion bioreactors, forced-aeration culture vessels, and automated aeroponic hardening systems) strictly where they evaluate ex vitro physiological competence, the alleviation of tissue culture-induced disorders (vitrotype reversal), and post-transplant survival.

Exclusion Criteria: (i) Conventional, open-field agronomic trials reporting macro-scale yield or general crop husbandry devoid of tissue culture preconditioning, physiological priming, or ex vitro acclimatization parameters; (ii) studies conducted on non-target species outside *S. tuberosum*; (iii) gray literature, conference proceedings lacking complete methodological data, and non-indexed academic theses.

### 2.3. Study Selection and Data Extraction

The initial database screening retrieved a cumulative total of 447 records (Scopus: 100; Web of Science: 23; ScienceDirect: 324). Following the removal of 85 overlapping duplicates using Mendeley Reference Manager (version 2.144.0, Elsevier), a refined corpus of 362 unique entries underwent title and abstract evaluation. To mitigate selection bias, this procedure was conducted independently by two investigators, with any discrepancies resolved through consensus arbitration involving a third senior reviewer.

**Figure 1:**
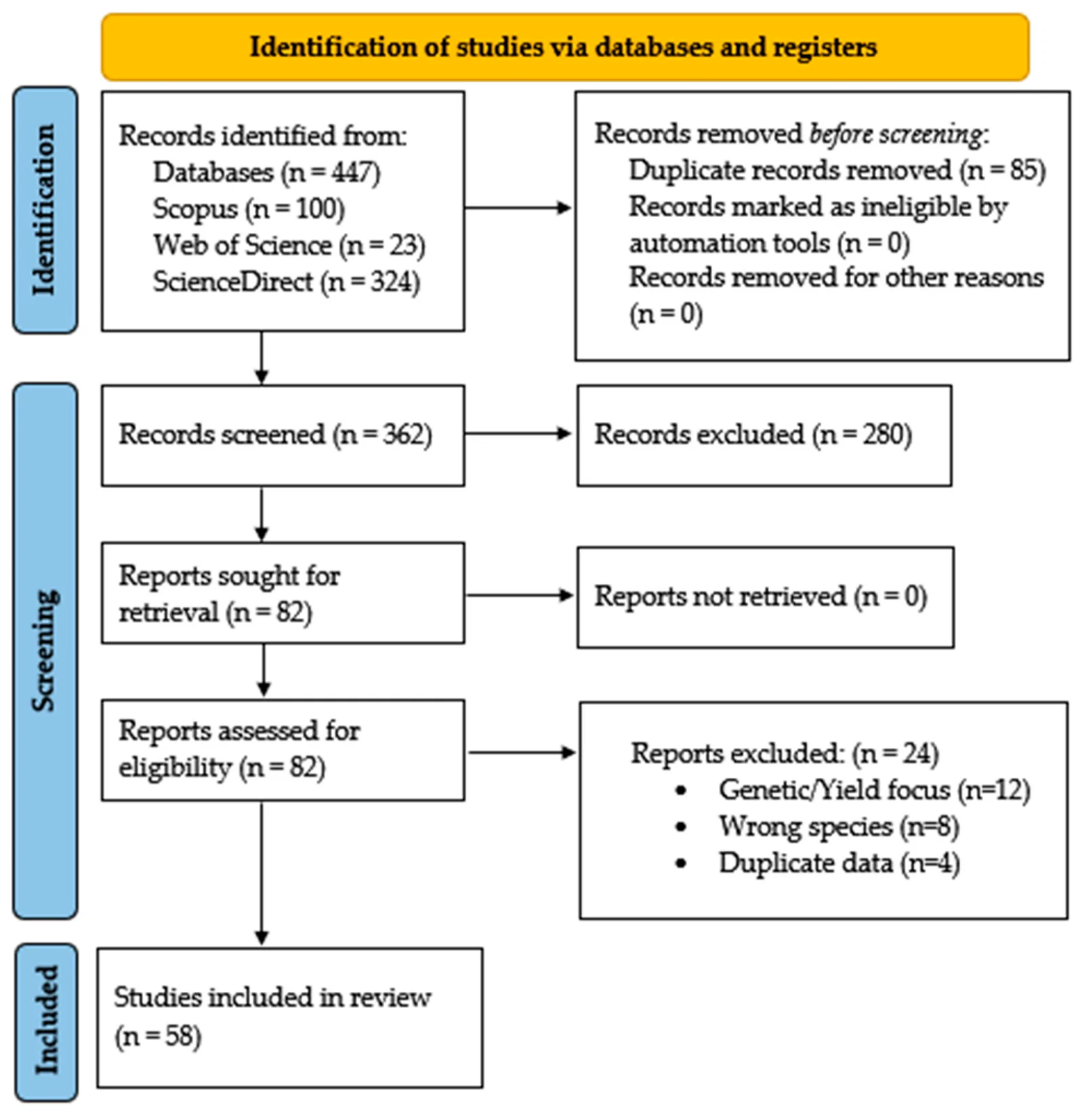
PRISMA 2020 flow diagram illustrating the literature search and study selection process for the review.

During this initial phase, 280 records were excluded as they failed to meet the molecular criteria or focused strictly on conventional propagation. Consequently, 82 full-text reports were sought for retrieval, and all 82 were successfully accessed and assessed for eligibility. Upon detailed appraisal, 24 studies were omitted for the following reasons: (i) an exclusive focus on genetic or yield traits devoid of molecular variables (*n* = 12); (ii) the employment of non-target biological models outside *S. tuberosum* (*n* = 8); (iii) dataset redundancy or the absence of discrete elicitation protocols (*n* = 4). Ultimately, 58 peer-reviewed empirical investigations satisfying all predefined physiological and micropropagation criteria from the initially retrieved 82 full-text reports were retained and incorporated into the narrative synthesis (Figure 1), while 24 studies were excluded based on objective scientific rationales (Supplementary Table S1). To ensure absolute methodological transparency in accordance with PRISMA 2020 guidelines, it is declared that the final bibliography contains 73 references. The 15 supplementary citations falling outside the reviewed cohort strictly correspond to the foundational theoretical framework and methodological guidelines utilized in the Introduction and Methods sections.

### 2.4. Quality Assessment and Risk of Bias

To evaluate the methodological quality and risk of bias of the included studies, evidence was systematically appraised by two independent reviewers using a custom 5-point checklist adapted for plant in vitro and molecular assays. The evaluation appraised five discrete diagnostic domains: (1) proper randomization of experimental units (D1); (2) stringent control and explicit documentation of in vitro and greenhouse microenvironmental parameters (D2); (3) adequacy of biological replication for transcriptomic and epigenetic assays (D3); (4) statistical reproducibility and deployment of advanced testing models (D4); (5) transparent, comprehensive outcome reporting to avoid selective publication bias (D5).

A complete evaluation matrix specifying domain performance for each study is provided in Supplementary Table S2. An elevated risk profile was defined as presenting non-compliance or severe reporting deficits across two or more domains, or substantial omission of critical microenvironmental parameters (D2). Overall, 7 out of the 58 included studies (12.1%) exhibited an elevated risk profile (predominantly due to incomplete reporting of in vitro microenvironmental parameters such as vapor pressure deficit or spectral irradiance), as summarized in Supplementary Table S2. These studies were retained solely to map broader qualitative trends across the literature, though their quantitative outcomes were interpreted with analytical caution.

### 2.5. Data Extraction Process

Data were systematically extracted by two independent reviewers using standardized templates, with any disagreements resolved by consensus or consultation with a third reviewer. Variables extracted included elicitor type/dosage, targeted transcriptomic markers (e.g., ROS-scavengers), epigenetic modifiers, and ex vitro survival rates.

### 2.6. Synthesis Methods and Heterogeneity

Due to substantial methodological variability across the retrieved literature specifically regarding cultivar genetic backgrounds, exogenous elicitor dosages, and disparate multi-omics platforms (e.g., RNA-seq versus targeted RT-qPCR, and MSAP versus bisulfite sequencing), a formal quantitative meta-analysis was precluded. Instead, a critical narrative synthesis approach was adopted.

The extracted data were systematically structured by clustering evidence into three primary domains of physiological competence: (1) epigenetic plasticity and in vitro stress memory; (2) transcriptomic reprogramming and defense signaling networks; (3) macroscopic morphogenic outcomes and ex vitro acclimatization success. This systematic categorization enabled the identification of converging molecular pathways despite experimental design heterogeneities.

## 3. Results

### 3.1. Epigenetic Plasticity, Stress Memory, and Clonal Fidelity In Vitro

The transition of *S. tuberosum* tissues into the in vitro environment imposes a profound morphogenic shock, triggering widespread tissue culture-induced variations modulated by multi-layered epigenetic mechanisms. Genome-wide profiling elucidates the critical role of histone modifications; specifically, histone methyltransferases (*StPRMTs*) and demethylases (*StHDMAs*) function as central epigenetic regulators modulating extreme abiotic stress responses and safeguarding cellular totipotency [16]. To systematically elucidate this regulatory plasticity during micropropagation and ex vitro hardening, the synthesized evidence within this domain is organized across four coordinated molecular layers:

First, DNA methylation dynamics operate as a primary regulatory tier during stress preconditioning. Genome-wide methylome profiling reveals that biotic elicitation, such as deoxynivalenol (DON) exposure, induces targeted DNA hypomethylation within transposable elements and stress-responsive loci, establishing an acquired epigenetic memory that broadens resistance networks without altering structural nucleotide sequences [17,18].

Second, post-translational histone modifications dynamically modulate chromatin compaction and cellular totipotency under physical constraints. Specifically, the dynamic recruitment of histone methyltransferases (*StPRMTs*) and histone deacetylases/demethylases (*StHDMAs*) fine-tunes gene expression under extreme thermal regimes to sustain basal developmental competence [16].

Third, non-coding RNA and post-transcriptional networks govern developmental competence and tuberization plasticity under adverse microenvironments. Small regulatory RNAs (sRNAs) actively repress *StSP6A* mRNA accumulation under heat stress, halting premature source–sink transitions [19], whereas exogenous ascorbic acid supplementation reverses this inhibition by restoring *StSP6A* expression [20]. These post-transcriptional adjustments are accompanied by the coordinated upregulation of foliar MEP and MVA pathway genes under elevated temperatures [21], as well as functional promoter activation of chalcone isomerase (*CHI*) directing anthocyanin secondary metabolism [22].

Fourth, chromatin architecture and biochemical stress-memory remodeling establish an essential physiological buffer against environmental shock. High-throughput RNA-seq demonstrates that during acute developmental and physical transitions—such as cryopreservation recovery—abiotic stress-responsive genes undergo extensive transcriptional reprogramming while fundamental housekeeping machinery remains functionally intact [23]. Concurrently, severe osmotic transitions modulate intracellular polyamine pools and antioxidant enzyme cascades, detoxifying reactive oxygen species (ROS) and reinforcing stress memory in vitro [24].

Ultimately, harnessing this molecular stress memory is a prerequisite for overcoming ex vitro transplant shock. Extended culture under artificial physical regimes triggers extensive proteomic adjustments; monochromatic red and blue light regimes differentially modulate photosynthetic carbon fixation and MAPK signaling proteins [25], while skotomorphogenesis upregulates specific cysteine protease inhibitors during sprout emergence [26]. In parallel, optimized clonal micropropagation protocols utilizing balanced hormonal regimes (BAP, IBA, and 24-epibrassinolide) preserve heterozygous features and guarantee pathogen-free tuberization [27].

Regarding the transgenerational kinetics and stability of elicitor preconditioning, the synthesized evidence confirms that elicitor-driven chromatin modifications are predominantly transient and reversible rather than permanent [17,27]. While targeted DNA hypomethylation and histone modifications provide a functional “stress memory” that primes defense networks during the acute ex vitro acclimatization window, these epigenetic marks undergo progressive resetting during subsequent vegetative propagation and tuber development cycles [17,27]. This programmed reversibility prevents permanent somaclonal divergence, thereby safeguarding true-to-type clonal fidelity across clonal generations [17,27]. While standard screening tools such as RAPD, ISSR, and SCoT markers validate structural genetic fidelity and ploidy stability [27], evaluating the exact kinetics of this transient epigenetic plasticity requires high-resolution methylation-sensitive amplification polymorphism (MSAP) or whole-genome bisulfite sequencing protocols (Table 1).

**Table 1:** Comparative overview of molecular, epigenetic interventions, and genetic fidelity assessments in *S. tuberosum* in vitro systems.

Reference	Focus Area	Intervention/Stress Condition	Molecular/Epigenetic Target	Key Quantitative & Molecular Findings
Mali & Zinta [16]	Abiotic Stress (Heat)	Heat exposure at 32 °C (day) and 27 °C (night)	Histone Modifiers	Identified 9 StPRMT and 11 StHDMA genes acting as central epigenetic regulators during thermotolerance.
Shi et al. [17]	Biotic Stress (Toxins)	Deoxynivalenol (DON) exposure at 0, 5, 20, 35, and 50 ng/mL	DNA Methylome	Targeted DNA demethylation within transposon regions establishes epigenetic memory for disease resistance.
Mir et al. [18]	Stress Resilience	Phytohormone application (Abscisic acid, jasmonic acid, salicylic acid, and ethylene)	Signaling Molecules	Demonstrated complex signaling crosstalk enhancing overall in vitro stress resilience networks.
Lehretz et al. [19]	Epigenetic & Post-transcriptional	Heat stress (Elevated temperatures)	sRNA/*SP6A* mRNA	Discovered a specific small regulatory RNA that represses *SP6A* mRNA accumulation, blocking tuberization under heat.
Lei et al. [20]	Transcriptomic Signaling	Exogenous Ascorbic Acid (1–5 mmol/L)	*StSP6A* gene expression	Exogenous AsA triggered tuberization by significantly up-regulating *StSP6A* during early microtuber formation.
Campbell et al. [21]	Heat Stress Transcriptomics	Moderately elevated temperatures	MEP and MVA pathway genes	Altered expression patterns of MEP and MVA genes corresponded to a six-fold increase in foliar solanesol.
Chen et al. [22]	Gene Expression & Promoter Analysis	Hormone induction	*CHI* promoter	Validated cis-regulatory elements and demonstrated *CHI* gene expression dynamics in tuber tissues.
Basu et al. [23]	Transcriptomic Abiotic Stress	Deep freezing (Cryopreservation)	RNA-seq/DEGs	Transcriptome profiling confirmed dynamic reprogramming of abiotic stress-responsive DEGs across regeneration stages, while essential housekeeping genes remained functionally stable during recovery.
Sajid & Aftab [24]	In vitro Osmotic Stress	Polyethylene glycol, sorbitol, and mannitol	Polyamines & Antioxidant Proteins	Demonstrated significant polyamine modulation to detoxify active oxygen species and enhance in vitro stress memory.
Xu et al. [25]	Proteomic Adaptation	Red and Blue LED light	Proteome/2982 DEPs	Identified 2982 differentially expressed proteins (DEPs) involved in photosynthesis and hormone signal transduction.
Grandellis et al. [26]	Transcript Profiling	Extended darkness during sprouting	Microarray/Cysteine protease	Transcript profiling revealed that cysteine protease inhibitors are significantly up-regulated in tuber sprouts.
Fattorini et al. [27]	Clonal Fidelity & Propagation	Sequential hormonal treatments (BAP, IBA, eBL)	Morphological & Cytological features	Validated an optimized in vitro clonal propagation method that maintains heterozygous features and ensures pathogen-free tuberization.

### 3.2. Transcriptomic Markers, Epigenetic Reprogramming, and Abiotic Stress Signaling Networks

The transition of *S. tuberosum* plantlets through in vitro micropropagation and subsequent ex vitro acclimatization exposes tissues to multifaceted abiotic constraints, predominantly osmotic fluctuations, hyperhydricity, and thermal stress. Managing these stresses requires the rapid reprogramming of transcriptomic and epigenetic signaling cascades rather than unprimed physiological acclimation. Transcriptomic evaluations demonstrate that pre-treating microplants with elevated thermal regimes (30/22 °C) significantly upregulates molecular chaperone families, including HSP20 and HSP90, alongside heat shock factors (HSFs) to safeguard proteostasis [28]. Concurrently, distinguishing ionic toxicity from pure osmotic adjustments is critical; in vitro microplants subjected to selective osmotic constraints adjust intracellular osmolyte pools through the coordinated induction of polyamines and antioxidant enzymes, maintaining cellular turgor without incurring ion-destructive penalties [29,30].

Transcriptomic profiling in liquid culture systems with controlled sorbitol application demonstrates that pure osmotic stress selectively activates drought-linked structural transcripts, elevating free proline pools over 1000-fold within 14 days [31]. At the chromatin level, abiotic constraints trigger profound architectural remodeling; cold exposure induces enhanced chromatin accessibility and the deposition of bivalent histone marks (H3K4me3 and H3K27me3) across active regulatory loci [32]. When microplants encounter hyperosmotic constraints in cell suspension cultures, mass spectrometry-based proteomics reveals that acute and stepwise transitions differentially reprogram nuclear chromatin organization and metabolic protein complexes [33]. This dynamic plasticity is further reinforced during darkness-induced microtuberization, where comprehensive RNA-seq profiling unveils the activation of ancestral introgression genes (such as *H3.2* and *GAPCP1*) alongside protein interaction networks governing carbon and sterol metabolism [34]. In parallel, biotic and abiotic priming via natural elicitors such as chitosan triggers extensive transcriptomic reprogramming, selectively inducing pathogenesis-related (*PR*) genes and activating phenylpropanoid defense networks prior to stress exposure [35]. These regulatory cascades operate in tight coordination with the DNA methylation and histone modification machinery, where DNA methyltransferases, demethylases [36], and Jumonji C (JmjC) domain-containing histone demethylase genes [37] exhibit dynamic, genotype-dependent expression across leaf and stolon tissues under high-temperature stress.

Downstream survival networks depend heavily on the activation of specialized transcription factors and kinase cascades. Crucially, evaluating the biological validity of these networks requires distinguishing descriptive transcriptomic profiling from direct functional genomic validation. While high-throughput RNA-seq and transcriptional profiling investigations [23,28,31,34,35,38] provide essential correlative inventories of stress-responsive regulons, targeted functional genomic studies establish direct causality through transgenic modification and biochemical validation: (i) Transcription Factor Overexpression: Transgenic overexpression of *StWRKY2* directly enhances stomatal closure and boosts enzymatic ROS scavenging, raising plantlet survival to 70% under severe dehydration [39]; similarly, ectopic expression of the cold-responsive regulon *ScCBF1* functionally lowers the freezing injury threshold (LT50 to −9.5 °C), outperforming native controls [40]. (ii) Kinase and Ubiquitination Assays: Overexpression of the brassinosteroid-signaling kinase *StBIN2* directly promotes shoot elongation under salinity stress [41], whereas transgenic upregulation of the U-box ubiquitin ligase *U-Box51* sustains elevated SOD and CAT enzyme activities to suppress lipid peroxidation under osmotic deficit [42]. (iii) Root Architecture Remodeling: Functional activation of the calcium-dependent protein kinase *StCDPK13* directly stimulates adventitious rooting, expanding root dry biomass by up to 2.8-fold under mannitol-induced osmotic shock [43]. Distinguishing these functionally validated nodes from descriptive transcriptomic candidates confirms that targeted signaling cascades can directly overcome in vitro recalcitrance and reduce transplant shock (Table 2).

**Table 2:** Transcriptomic markers, epigenetic reprogramming, and abiotic stress signaling hubs in *S. tuberosum* in vitro preconditioning systems.

Reference	Focus Area	Intervention/Stress Condition	Molecular/Transcriptomic Target	Key Quantitative & Molecular Findings
Alhaj Hamoud et al. [28]	Heat Stress	Elevated temperatures (30/22 °C)	Multi-omics/Chaperone families	Identified 3617 DEGs; severe heat shock significantly upregulated HSP20, HSP90, and HSF chaperone networks.
Hamooh et al. [29]	Osmotic & Salt Stress	150 mM mannitol and 20 mM LiCl	Antioxidant and Phenolic pathways	Cultivar-specific antioxidant and phenolic capacity preserved cellular membrane integrity under osmotic constraints.
Wellpott et al. [30]	In vitro Osmotic Stress	Liquid culture with gradual sorbitol	Transcriptional profiling/RT-qPCR	Confirmed strong upregulation of drought-linked transcripts and a 1093-fold elevation in sorbitol accumulation at day 14.
Jing et al. [31]	In vitro Salt Stress	In vitro salinity stress (200 mM NaCl)	RNA-seq/TF-hub genes (*StGLK014720*)	Identified 8383 DEGs; confirmed *StGLK014720* transactivates promoters of structural genes *StAST021010* and *StAST017480* driving proline biosynthesis.
Zeng et al. [32]	Epigenetic Cold Stress	Cold exposure (chilling regime)	Chromatin accessibility/Bivalent histones	Demonstrated enhanced chromatin accessibility and deposition of bivalent marks (H3K4me3 and H3K27me3) activating cold-responsive genes.
Cappetta et al. [33]	Osmotic Stress	Stepwise osmotic constraint in suspension	Proteome/Chromatin networks	High-throughput proteomics confirmed that osmotic transitions reprogram nuclear chromatin structure and metabolic cascades.
Valencia-Lozano et al. [34]	Microtuberization Stress	Darkness-induced in vitro tuberization	RNA-seq/PPI Network	Mapped 404 high-confidence genes; elucidated transcriptomic hubs coordinating carbon partitioning, sterol synthesis, and stress tolerance.
Lemke et al. [35]	Elicitor Priming	Chitosan application in vitro	RNA-seq/Defense transcripts	Transcriptome profiling revealed wide activation of *PR* genes and phenylpropanoid secondary metabolism pathways.
Dutta et al. [36]	Heat/Epigenetic Interplay	High-temperature stolon induction	DNA methyltransferases & demethylases	Revealed coordinated interplay between DNA methyltransferases (StDMTs) and demethylases (StDMLs) regulating tuberization genes under heat.
Mali et al. [37]	Epigenetic Heat Response	High temperature (leaf vs. stolon)	JmjC Histone Demethylases	Expression profiling of Jumonji C (JmjC) domain-containing genes revealed tissue-specific epigenetic tuning during heat acclimation.
Jin et al. [38]	Drought Stress	Polyethylene glycol simulated drought	StDof Transcription Factors	Mapped 36 StDof genes; identified 5 StCDFs functioning as initial cascade initiators under dehydration.
Shahzad et al. [39]	Dehydration Resilience	15-day water deprivation	StWRKY2 Transcription Factor	StWRKY2 overexpression increased plantlet survival from 17% (WT) to 70% by augmenting stomatal closure and ROS scavenging.
Li et al. [40]	Freezing Tolerance	Low-temperature chilling regime	ScCBF1/StCBF1 regulons	Ectopic ScCBF1 expression lowered the LT50 threshold to −9.5 °C, outperforming native StCBF1 (−6.0 °C) and wild-type controls.
Liu et al. [41]	Salinity Preconditioning	200 mM NaCl in vitro stress	StBIN2 Kinase	StBIN2 overexpression conferred higher tolerance, improving plantlet height by 1.13-fold compared to non-transgenic controls.
Wei et al. [42]	Osmotic Stress Memory	20% PEG-6000 simulated drought	U-Box51 Ubiquitin Ligase	U-Box51 positively maintained high SOD and CAT enzyme activities, drastically suppressing malondialdehyde (MDA) accumulation.
Bi et al. [43]	Osmotic Resilience	200 mM mannitol application	StCDPK13 Kinase	StCDPK13 activation stimulated adventitious rooting, increasing dry root biomass by 2.1- to 2.8-fold under severe osmotic constraint.

### 3.3. Exogenous Elicitors, Biotic Priming, and Metabolic Regulation

In vitro propagation platforms provide a highly controlled environment to investigate the application of exogenous elicitors, which operate as critical modulators of plant metabolic pathways and stress resilience [44]. Exogenous supplementation with signaling phytohormones, particularly jasmonic acid (JA) at 1.0 µM, significantly accelerates in vitro propagation rates, increasing shoot length up to 15.13 cm and fresh weight to 649.38 mg under specific long-day photoperiods [45]. Furthermore, abscisic acid (ABA), particularly when interacting synergistically with 80 g/L sucrose, assumes a vital role: sucrose drives microtuber development (up to 6.2 microtubers/shoot), while 0.5 mg/L ABA prolongs storage viability by delaying germination to 15–18 days, an essential prerequisite for germplasm conservation [46]. The utilization of alternative aromatic cytokinins like meta-Topolin (mT) has emerged as an effective strategy to optimize *S. tuberosum* micropropagation and mitigate in vitro recalcitrance. In potato cultivars such as Tezu local, MS media supplementation with 0.5 mg/L mT stimulates multiple shoot induction (up to 5.0 shoots per explant), while simultaneous integration of 1.0 mg/L mT with 3.0 mg/L IBA establishes a mono-phasic regeneration system supporting robust adventitious rhizogenesis (20.7 roots/shoot). This hormonal preconditioning preserves cellular ploidy stability and secures 100% survival during primary ex vitro acclimatization in cocopeat substrates [47]. Additionally, the foliar application of signaling compounds, specifically monopotassium phosphate (MPPh) and potassium silicate (PSi), directly impacts assimilate remobilization, effectively increasing tuber yield by 60% and 45%, respectively, while elevating hydrogen peroxide levels to accelerate late-season leaf senescence [48].

Beyond primary growth induction, chemical elicitors trigger extensive metabolomic crosstalk and dictate intricate structural adaptations [49]. In *S. tuberosum* micropropagation, chemical nano-elicitation directly alleviates osmotic and transplant-induced constraints; supplementing MS media with nano-formulations of salicylic acid (SA) and benzoic acid (BA) (0.50–0.75 mM) enhances stem elongation and adventitious root proliferation in nodal explants, counteracting PEG-induced water deficit and preserving plantlet vigor [50]. Concurrently, to evaluate ex vitro agronomic translation in pot-grown *Solanum tuberosum* cv. Favorita under controlled greenhouse conditions, foliar application of 200 µmol/L methyl jasmonate (MeJA) increased tuber yield by up to 242.4% while simultaneously reshaping the phyllosphere epiphytic bacterial consortia through fatty acid-mediated niche specialization, enriching stress-resistant Proteobacteria [51]. These physiological and agronomic responses are intrinsically coupled with endogenous hormonal dynamics; optimized ultra-high-performance liquid chromatography (UHPLC) profiling of salicylic acid, indole-3-acetic acid (IAA), ABA, and JA demonstrates distinct tissue-specific hormonal fluctuations that functionally regulate apical dominance, stolon development, and microtuber dormancy release [49].

At the molecular level, the synthesis of secondary metabolites is tightly regulated; R2R3-MYB transcription factors interact via a feedback loop with 120 mM sucrose and specific microRNAs to fine-tune flavonol biosynthesis. For instance, *StmiR858* overexpression represses *MYB12A* by 72%, effectively decreasing flavonol concentrations by approximately 57% [52]. Similarly, microtuberization under total darkness actively engages a 15-gene module within the mevalonate (MVA) pathway, where *HMGR3* is upregulated ~4-fold to stimulate the biosynthesis of phytosterols from 8% sucrose media [53]. Furthermore, hormonal and carbohydrate priming interactions critically govern in vitro morphogenesis; in *S. tuberosum* cv. Burin, the synergistic supplementation of 80 g/L sucrose with 2.0 mg/L each of BAP and kinetin optimizes branching dynamics and maximizes microtuber biomass [54].

Metabolic regulation is equally decisive in managing systemic stress and biotic interactions. Environmental constraints induce severe metabolic shifts; a meta-analysis of 223 drought-responsive metabolites confirms the consistent upregulation of protective osmolytes like trehalose and L-proline among 172 distinct metabolites in potato [55]. Under severe cold stress (−4 °C for 7 days), key enzymatic pathways are directly modulated; the upregulation of *StPAL* and *StGAD* pathways accelerates the accumulation of cinnamic acid and GABA, limiting leaf damage to 12.2% in resistant cultivars [56]. Simultaneously, ensuring clonal stability during hormonal induction is paramount; combining specific auxin–cytokinin ratios (0.01 mg/L NAA with 0.25 mg/L kinetin) promotes multiple shoot proliferation and 100% in vitro rhizogenesis while preserving strict genetic fidelity across regenerated plantlets [57]. Biotic priming introduces a complementary dimension to this regulation, bridging in vitro protocols with field-level agronomy. The inoculation of in vitro potato microplants with plant-growth-promoting rhizobacteria (PGPR), such as *Azospirillum* strains (producing up to 87.3 µg/mL of IAA), significantly promotes ex vitro survival, a response heavily driven by this bacterial IAA production [44]. Furthermore, optimizing vessel physical microenvironments and sucrose nutrition establishes superior autotrophic competence; in elite *S. tuberosum* cultivars, full-strength MS medium supplemented with 3% sucrose yields 100% in vitro rhizogenesis, achieving shoot multiplication rates up to 42.3-fold and securing 80–90% ex vitro survival during greenhouse acclimatization [58]. Ultimately, integrating these advanced metabolic insights with the nutritional profiling of wild diploid species, such as *Solanum okadae*, unlocks superior traits—including 18.47 g/100g carbohydrates, 30.10 g/kg potassium, and the rare presence of Vitamin B12 (0.28 mg/100g)—establishing a robust genetic pathway for future biofortification [59] (Table 3).

**Table 3:** Elicitors, biotic priming agents, and metabolic regulation in *S. tuberosum*.

Reference	Focus Area	Elicitor/Biotic Agent	Key Quantitative & Molecular Findings
Kargapolova et al. [44]	Biotic Priming	Azospirillum (PGPR)	Bacterial IAA production (up to 87.3 µg/mL) from *A. brasilense* Sp245 strongly enhanced potato microplant ex vitro survival (96%).
Kumlay [45]	Micropropagation	Jasmonic Acid (JA)	1.0 µM JA significantly accelerated multiplication, achieving maximum shoot length (15.13 cm) and fresh weight (649.38 mg).
Shukla et al. [46]	Dormancy Regulation	ABA & Sucrose	80 g/L sucrose drove microtuber development (6.2/shoot), while 0.5 mg/L ABA extended storage viability by delaying germination to 15–18 days.
Bandyopadhyay et al. [47]	Micropropagation & Acclimatization	meta-Topolin (mT)	In *S. tuberosum* cv. Tezu local, 0.5 mg/L mT induced 5.0 shoots/explant; combining 1.0 mg/L mT with 3.0 mg/L IBA yielded 20.7 roots/shoot, unaltered ploidy, and 100% ex vitro survival.
Hosseinian et al. [48]	Leaf Senescence	Signaling Compounds	MPPh and PSi applications increased total tuber yield by 60% and 45% respectively, elevating hydrogen peroxide to induce leaf senescence.
Zhao et al. [49]	Endogenous Hormones	SA, IAA, ABA, JA	Optimized UHPLC (100% acetonitrile, 25 min ultrasound) mapped hormone fluctuations during dormancy breaking with RSD < 10%.
Alowaiesh et al. [50]	Chemical Nano-Elicitation	Salicylic Acid & Benzoic Acid NPs	In *S. tuberosum* nodal cuttings, 0.50–0.75 mM SA and BA nanoparticles significantly enhanced root number, root length, and shoot elongation under 30% PEG-simulated drought stress.
Fang et al. [51]	Microbiome Regulation	Methyl Jasmonate (MeJA)	200 µmol/L MeJA delayed senescence, reduced MDA by 35.7%, and increased yield by up to 242.4% while restructuring epiphytic bacteria.
Lin et al. [52]	Flavonol Biosynthesis	Sucrose & R2R3-MYB	120 mM sucrose induced flavonols; StmiR858 overexpression repressed MYB12A by 72% and decreased flavonol concentrations by 57%.
Herrera-Isidron et al. [53]	Microtuberization	Phytosterols	Darkness engaged a 15-gene MVA network; HMGR3 upregulated ~4-fold to synthesize phytosterols using 8% sucrose and 10 mg/L 2-iP.
Ibrahim et al. [54]	Micropropagation & Tuberization	BAP, Kinetin & Sucrose	In *S. tuberosum* cv. Burin, 80 g/L sucrose combined with 2.0 mg/L BAP and KIN maximized microtuber yield (10 tubers/jar) and mean tuber weight (1.07 g).
Mutanda et al. [55]	Drought Stress	Osmolytes (Proline/Trehalose)	Meta-analysis of 223 responsive metabolites identified trehalose and L-proline among 172 distinct metabolites crucial for osmotic adjustment in potato.
Li et al. [56]	Cold Stress	GABA & Cinnamic Acid	StPAL and StGAD upregulation during −4 °C stress accumulated cinnamic acid and GABA, limiting leaf damage to 12.2% in resistant cultivars.
Rout et al. [57]	Clonal Fidelity & Organogenesis	NAA, IBA & Kinetin	Auxin–cytokinin combinations induced 10.1 shoots/explant and 100% rooting in cv. Kufri Lima; RAPD profiling confirmed monomorphic banding and clonal fidelity.
Patel et al. [58]	Clonal Propagation & Acclimatization	MS Media & Sucrose Priming	In *S. tuberosum* cultivars (KCS, KFM, KFS), 3% sucrose yielded 100% rooting, 35.0–42.3-fold shoot proliferation, and 80–90% ex vitro acclimatization survival.
Hundare [59]	Nutritional Profiling	Metabolic Comparison	Diploid *S. okadae* exhibited 18.47 g/100g carbohydrates, 30.10 g/kg Potassium, and unique presence of Vitamin B12 (0.28 mg/100g).

### 3.4. Technological Scalability, Bioreactor Systems, and Ex Vitro Acclimatization Platforms

Translating in vitro preconditioning protocols from experimental bench-scale systems to commercial seed potato (*S. tuberosum*) production requires scalable propagation platforms capable of supporting vigorous morphogenic development while preventing tissue culture-induced abnormalities [60]. The deployment of temporary immersion bioreactors (TIBs) has transformed large-scale micropropagation by ensuring periodic gas exchange and uniform liquid nutrient contact, effectively doubling shoot proliferation rates while mitigating hyperhydricity and mechanical shear stress [60]. To resolve operational constraints associated with media formulation, nutrient-use optimization strategies demonstrate that substituting standard Murashige and Skoog (MS) macro- and micronutrients with refined low-cost mineral complexes maintains micropropagation performance and post-flask vigor [61]. Parallel culture innovations utilizing disposable culture bags equipped with forced ventilation systems provide an aerated, scalable environment capable of yielding 100 to 300 uniform microtubers per 8 L unit, directly preventing container asphyxiation [62]. Furthermore, incorporating biological inputs such as vermicompost into post-flask substrates enhances nutrient uptake and shoot-to-root biomass partitioning, yielding vigorous seed tubers (up to 173.12 g/plant) with superior post-transplant performance [63]. Securing successful ex vitro transition relies increasingly on automated soilless technologies that bridge laboratory micropropagation and commercial greenhouse acclimatization [64]. Aeroponic systems provide high rhizospheric aeration and precise misting intervals, which eliminate the hydraulic shock common to soil potting substrates while drastically reducing water requirements to 1/10–1/30 of conventional practices and generating up to 800 tubers/m^2^ over a 5-month period [64]. Integrating automated Internet of Things (IoT) sensor architectures into high-density aeroponic chambers directly resolves hardening microclimate instability; by coupling real-time telemetry of rhizospheric temperature, root-zone relative humidity, and electrical conductivity with automated feedback actuators, these systems dynamically modulate ultrasonic misting intervals, preventing hydraulic collapse and achieving propagation densities exceeding 900 mini-tubers/m^2^ [65]. In parallel, substrate inoculation with beneficial fungal biocontrol agents, such as *Trichoderma harzianum* (0.5 g/L), enhances root colonization and suppresses transplant-associated stress, securing high plantlet vigor and elevating tuber production up to 57.13 t/ha [66]. Evaluating alternative management regimes confirms that balanced nutrient delivery directly modulates tuber secondary metabolism, yielding up to 18% higher total phenolics and providing physiological protection against oxidative damage during storage and subsequent sprouting [67]. Domestically optimized micropropagation platforms provide robust commercial multiplication frameworks for clean seed supply [68], ensuring the effective preservation of clonal stability and agrobiodiversity against environmental pressures [69].

**Figure 2:**
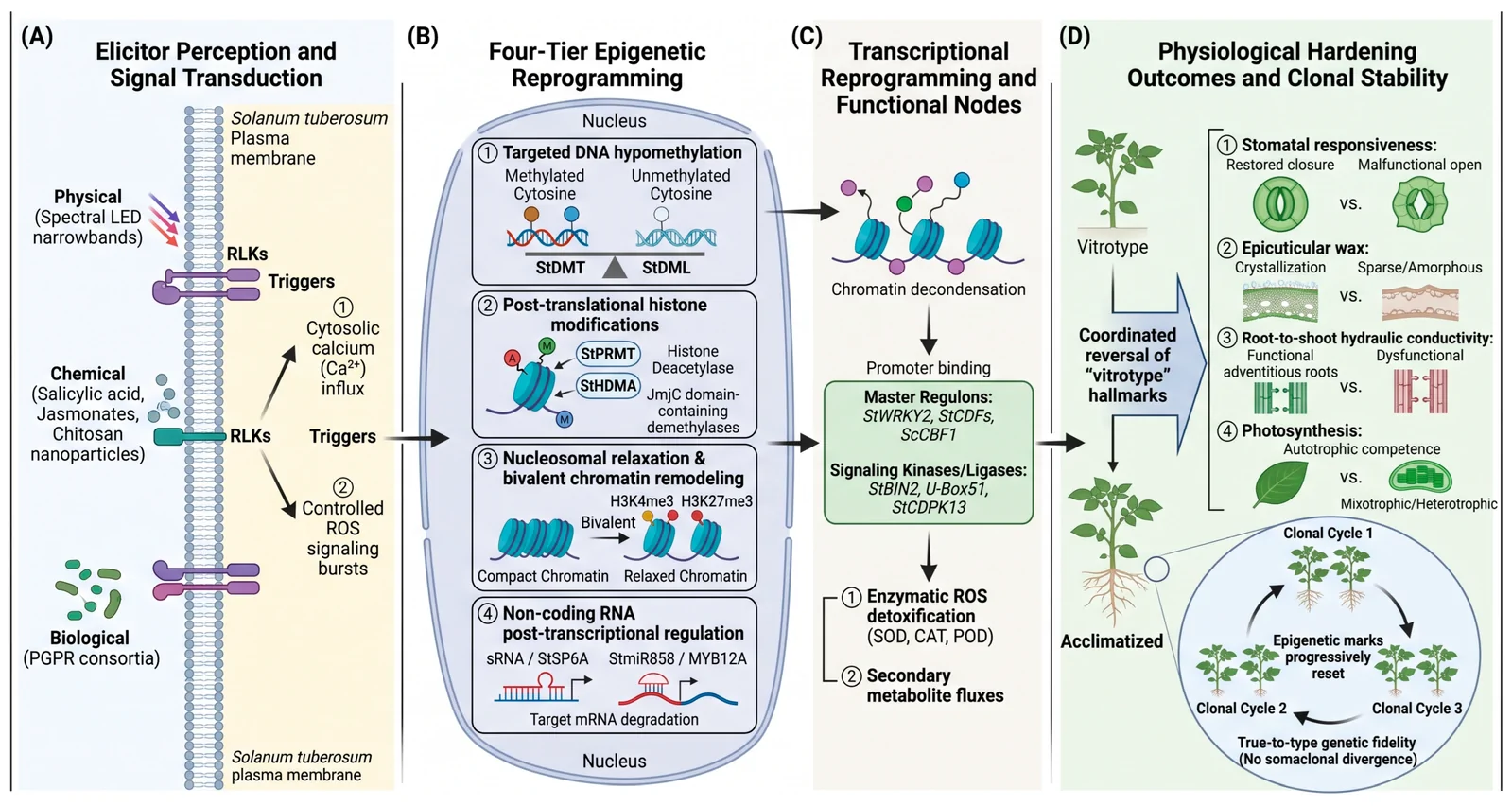
Integrated mechanistic model of elicitor perception, epigenetic reprogramming, and ex vitro acclimatization in *Solanum tuberosum* L. (**A**) Elicitor Perception and Signal Transduction: Plasma membrane perception of physical (LED narrowbands), chemical (salicylic acid, jasmonates, chitosan nanoparticles), and biological (PGPR consortia) elicitors by receptor-like kinases (RLKs), triggering rapid cytosolic calcium (${\mathrm{Ca}}^{2+}$) influxes and controlled reactive oxygen species (ROS) signaling bursts. (**B**) Four-Tier Epigenetic Reprogramming: Dynamic nuclear machinery organized across: (1) targeted DNA hypomethylation governed by *StDMT*/*StDML* transcriptional balance [17,36]; (2) post-translational histone modifications mediated by *StPRMT*, *StHDMA*, and JmjC domain-containing demethylases [16,37]; (3) nucleosomal relaxation and bivalent chromatin remodeling (H3K4me3/H3K27me3) establishing transcriptional accessibility [32,33]; (4) non-coding RNA post-transcriptional regulation (*sRNA*/*StSP6A*, *StmiR858*/*MYB12A*) [19,52]. (**C**) Transcriptional Reprogramming and Functional Nodes: Chromatin decondensation enables rapid promoter binding by master regulons (*StWRKY2*, *StCDFs*, *ScCBF1*) and signaling kinases/ligases (*StBIN2*, *U-Box51*, *StCDPK13*), orchestrating enzymatic ROS detoxification (SOD, CAT, POD) and secondary metabolite fluxes [38,39,40,41,42,43]. (**D**) Physiological Hardening Outcomes and Clonal Stability: Coordinated reversal of the six hallmarks of the “vitrotype” disorder, resulting in restored stomatal closure responsiveness, epicuticular wax crystallization, functional adventitious root-to-shoot hydraulic conductivity, and autotrophic photosynthetic competence [4,5,6,43]. Epigenetic marks progressively reset during successive clonal cycles (Clonal Cycles 1–3), ensuring robust ex vitro survival while preserving true-to-type genetic fidelity without somaclonal divergence [17,27,57]. Abbreviations: Ca^2+^, calcium ion; CAT, catalase; JmjC, Jumonji C domain-containing protein; MAPK, mitogen-activated protein kinase; ncRNA, non-coding RNA; PGPR, plant growth-promoting rhizobacteria; POD, peroxidase; RLKs, receptor-like kinases; ROS, reactive oxygen species; SOD, superoxide dismutase; StBIN2, *Solanum tuberosum* brassinosteroid-insensitive 2; StCDFs, cycling Dof factors; StCDPK13, calcium-dependent protein kinase 13; StDML, DNA demethylase; StDMT, DNA methyltransferase; StHDMA, histone deacetylase; StPRMT, protein arginine methyltransferase; StWRKY2, WRKY transcription factor 2.

At the commercial scale, the ultimate efficacy of in vitro preconditioning systems is evaluated by their capacity to produce high-performing, certified seed stocks ready for widespread deployment [70]. Targeted micronutrient pre-treatments, such as specialized zinc applications, enhance basal enzymatic activity and stress resilience, establishing favorable resource-use ratios during downstream vegetative development [71]. Multi-year clonal reproduction schemes underscore that accelerated in vitro micropropagation protocols successfully yield virus-free, uniform seed tubers that sustain robust field emergence and high commercial productivity (reaching 56 t/ha in cv. Daryonka) [72]. Finally, integrating physiologically based crop models allows researchers to correlate in vitro preconditioning parameters and microplant quality with post-acclimatization performance under variable temperature and moisture conditions [73]. Combining molecular elicitation, automated bioreactor culture, and aeroponic hardening provides an integrated biotechnological pipeline to produce pathogen-free, stress-resilient seed potato stocks (Figure 2). Beyond biophysical yield metrics, addressing the systemic sustainability of *S. tuberosum* micropropagation requires evaluating its environmental footprint. Standard laboratory pipelines remain energy-intensive due to continuous photoperiod illumination and HVAC thermoregulation, alongside substantial solid waste generated by single-use plastic vessels. However, transitioning to automated temporary immersion bioreactors (TIBs) and downstream aeroponic platforms dramatically enhances eco-efficiency; liquid-phase automation maximizes vessel reusability, minimizes nutrient waste, and curtails water consumption to 1/10–1/30 of conventional nursery requirements while eliminating agrochemical runoff [60,64]. Coupled with energy-efficient LED luminaires, these scalable platforms establish a resource-efficient biofactory paradigm for sustainable clean seed supply (Table 4).

**Table 4:** Technological platforms, bioreactor systems, and sustainable propagation strategies in *S. tuberosum*.

Reference	Focus Area	Technological/Biological Platform	Key Quantitative & Morphogenic Findings
Lorenzo et al. [60]	Bioreactor Micropropagation	Temporary Immersion Bioreactors (TIBs)	Doubled shoot multiplication rates, optimized nutrient contact, and significantly minimized vitrification.
Peña-Rojas et al. [61]	Media Optimization	Alternative Nutrient Complexes	Validated alternative nutrient formulations in liquid and semi-solid systems without compromising plantlet vigor.
Mamiya et al. [62]	Culture Vessels & Aeration	Plastic Culture Bags with Forced Aeration	Provided high-aeration vessels yielding 100–300 uniform microtubers per 8 L container, preventing asphyxiation.
Boubaker et al. [63]	Substrate Priming	Biofertilizer/Vermicompost Integration	Enhanced in vitro microtuber sprouting, root development, and tuber biomass (173.12 g/plant).
Tunio et al. [64]	Soilless Acclimatization	Aeroponic Rooting Systems	Reduced water usage by 90–97% while supporting superior root aeration and producing 800 tubers/m^2^.
Tang et al. [65]	Automated Scaling	IoT-Monitored Aeroponic Chambers	Accelerated rapid clonal propagation to >900 mini-tubers/m^2^ via automated misting and environmental control.
Pelaez & Ttacca [66]	Microbial Hardening	*Trichoderma harzianum* Inoculation	Application of 0.5 g/L *T. harzianum* stimulated adventitious rooting and protected plantlets during hardening.
Djaman et al. [67]	Metabolic Quality	Controlled Nutrition Management	Modulated secondary metabolism, decreasing nitrate content by 34% and increasing phenolic defenses by 18%.
Sidibe et al. [68]	Clonal Multiplication	In Vitro Micropropagation Pipelines	Established robust, high-frequency seed production pipelines ensuring the sanitation of indigenous germplasm.
Villa [69]	Germplasm Conservation	Integrated Tissue Culture Systems	Evaluated systematic clonal conservation strategies to maintain genetic diversity and counter production bottlenecks.
Low et al. [70]	Large-scale Delivery	Scaling Clean Seed Technologies	Demonstrated scaling readiness of sanitized, biofortified clonal root and tuber crops across diverse regions.
Nagar et al. [71]	Micronutrient Priming	Zinc Sulphate Fortification	Combined basal (12.5 kg/ha) and foliar (0.2%) Zn application enhanced antioxidant enzyme competence and vigor.
Nikolaeva et al. [72]	Clonal Sanitation	Accelerated In Vitro Reproduction	Validated a multi-year biotechnological scheme for virus eradication, ensuring clonal uniformity and high yield.
Ahmed et al. [73]	Predictive Modeling	Process-Based Growth Models	Applied computational modeling to evaluate environmental factors influencing vegetative and tuber development.

## 4. Discussion

### 4.1. Mechanistic Convergence: Transcriptomic Defense and Epigenetic Plasticity

The systematic synthesis of the 58 included investigations reveals that mitigating transplant shock in micropropagated *Solanum tuberosum* L. requires coordinated, multi-layered cellular reprogramming rather than unprimed acclimation [1,3]. While our previous systematic review in *Horticulturae* [14] characterized media formulation, spectral quality, and bio-priming protocols, the present synthesis establishes the underlying molecular hierarchy governing in vitro competence. Physical elicitors and chemical priming agents bridge chromatin architecture with defense transcription networks [12,13]. Specifically, transient DNA hypomethylation [17] and histone modifications mediated by StPRMT, StHDMA [16], and Jumonji C (JmjC) domain demethylases [37] relax chromatin accessibility [32]. This permissive epigenetic state facilitates the rapid transcriptional induction of core transcription factors, including StWRKY2 [39] and the 36-member StDof family [38]. Downstream, these regulons orchestrate enzymatic ROS-scavenging cascades (SOD, CAT, POD) [24,42] and phenylpropanoid secondary metabolism [35], buffering microplants against lethal oxidative burst during ex vitro transfer [6,8]. Crucially, in line with transgenerational kinetics, this elicitor-driven chromatin remodeling operates as a transient physiological “stress memory” during the acute hardening window rather than inducing permanent somaclonal divergence, resetting during subsequent tuber reproduction cycles to safeguard true-to-type clonal fidelity [17,27].

To synthesize these intersecting pathways into a predictive framework, we propose an integrated multi-level mechanistic model delineating the biological continuum from in vitro perception to ex vitro phenotypic competence (Figure 2): (i) Plasma Membrane Perception and Early Signaling: Physical, chemical, and biological elicitors are recognized by membrane-associated receptor-like kinases and pattern recognition receptors [9,11], initiating rapid cytosolic Ca^2+^ influxes and transient, controlled reactive oxygen species (ROS) signaling bursts. (ii) Epigenetic Chromatin Decondensation: These secondary messengers trigger the downregulation of DNA methyltransferases alongside the recruitment of histone demethylases (StHDMA, JmjC) and the deposition of permissive bivalent marks (H3K4me3/H3K27me3) [16,32,36,37], relaxing heterochromatin compaction at stress-responsive loci. (iii) Transcriptional Cascade Activation: This open chromatin architecture facilitates rapid promoter binding by master regulons, predominantly StWRKY2 and the StDof/StCDF family [38,39], which directly upregulate enzymatic antioxidant systems (SOD, CAT, POD) and stress-protective kinases (StCDPK13, StBIN2) [41,42,43]. (iv) Physiological Manifestation and Vitrotype Resolution: This coordinated molecular cascade directly drives the functional recovery of stomatal closure, accelerates epicuticular wax crystallization, expands root-to-shoot hydraulic conductance, and establishes autotrophic photosynthetic competence [4,5,6,43], effectively preconditioning microplants to overcome transplant shock while preserving true-to-type clonal fidelity [27,57].

### 4.2. Physiological Reversal of the Vitrotype Phenomenon

A fundamental contribution of elicitor preconditioning is the direct alleviation of the morpho-physiological abnormalities defining the “vitrotype” disorder [4,5]. Under standard micropropagation, continuous immersion in water-saturated atmospheres and constant exogenous sucrose suppresses normal photosynthetic differentiation and ABA signaling [4,46]. Chemical priming with signaling elicitors restores endogenous ABA and auxin balances [44,49], driving functional stomatal closure mechanisms [39] and promoting precocious cuticular wax crystallization [6,29]. Furthermore, hormonal and nano-scale elicitation, such as meta-Topolin regimes [47] and salicylic/benzoic acid nanoparticles [50], stimulates adventitious rhizogenesis, establishing functional vascular connections essential for hydraulic conductance when plantlets transition from heterotrophic in vitro media to autotrophic greenhouse substrates [1,58].

### 4.3. Scalability, Environmental Footprint, and Sustainable Bioprocesses

Commercial implementation of preconditioning protocols requires scalable cultivation vessels capable of minimizing the environmental footprint of tissue culture facilities [60,70]. Conventional micropropagation systems incur high operational costs and heavy environmental burdens due to continuous artificial lighting, thermal regulation, and single-use plastic disposal [61,62]. As demonstrated across the included technological corpus, temporary immersion bioreactors (TIBs) substantially improve eco-efficiency by optimizing intermittent nutrient contact, eliminating hyperhydricity, and doubling shoot multiplication rates [60]. Complementarily, forced-aeration culture bags resolve container asphyxiation during microtuber development [62], while automated aeroponic chambers reduce water consumption by 90–97% compared to soil potting mixes [64]. Integrating Internet of Things (IoT) telemetry networks into aeroponic hardening modules [65] enables real-time sensing of rhizospheric temperature and vapor pressure deficit, triggering automated closed-loop misting adjustments that buffer plantlets against abrupt microclimatic fluctuations and ensure reproducible acclimatization without chemical fungicides [63,66].

### 4.4. Review Limitations and Risk of Bias Considerations

In accordance with PRISMA 2020 guidelines, several methodological limitations must be considered. First, high experimental heterogeneity across cultivar genetic backgrounds (ranging from commercial tetraploids like cv. Atlantic, Desiree, and Favorita to indigenous diploid accessions) [29,51,59], elicitor formulations, and exposure windows precluded a formal quantitative meta-analysis [30]. Second, our methodological appraisal identified that 12.1% of the synthesized studies (7 of 58) exhibited an elevated risk of bias, primarily stemming from incomplete reporting of baseline microenvironmental parameters (such as photosynthetic photon flux density and vapor pressure deficit) [26,31,35,61]. Third, the review was bounded by language criteria (English-language peer-reviewed articles) and the exclusion of non-indexed grey literature. Finally, while immediate in vitro stress memory is well documented, long-term multi-generational epigenomic stability and longitudinal field-yield assessments following elicitor withdrawal remain scarce [27,57,72]. Future investigations must prioritize standardized multi-omics frameworks, whole-genome bisulfite sequencing (WGBS), and life-cycle environmental assessments to consolidate elicitor preconditioning within sustainable seed potato production systems [70,73].

## 5. Conclusions

This systematic review synthesized evidence from 58 peer-reviewed studies retrieved across Scopus, Web of Science (Core Collection), and ScienceDirect spanning January 2010 to August 2026, establishing a comprehensive mechanistic framework for elicitor-driven preconditioning during *Solanum tuberosum* L. micropropagation. The collective findings demonstrate that successfully overcoming ex vitro transplant shock depends on an orchestrated, tripartite preconditioning continuum: (i) an elicitor-triggered transcriptomic reprogramming that upregulates enzymatic antioxidant defenses and reactivates defense cascades; (ii) a permissive epigenetic landscape governed by transient chromatin remodeling; (iii) scalable biofactory platforms (temporary immersion bioreactors and automated aeroponics) providing the precise microenvironmental control required to translate molecular priming into phenotypic vigor at commercial scales.

At the morpho-physiological level, physical, chemical, and biological elicitors directly reverse the classical hallmarks of the “vitrotype” disorder by restoring stomatal responsiveness, accelerating epicuticular wax crystallization, and stimulating functional adventitious root-to-shoot vascular connections. At the translational scale, integrating precision molecular preconditioning with scalable, automated bioreactor technologies—predominantly Temporary Immersion Bioreactors (TIBs) and aeroponic modules—dramatically improves resource eco-efficiency by cutting water consumption to a fraction of conventional potting nursery requirements, eliminating agrochemical runoff, and reducing container-related plastic waste. Notwithstanding these translational advances, the conclusions of this systematic review must be interpreted within the context of specific literature limitations: marked protocol and phenotypic heterogeneity across diverse potato genotypes (commercial tetraploids versus native accessions), a prominent deficit in high-resolution whole-genome bisulfite sequencing (WGBS) datasets quantifying base-resolution cytosine methylation dynamics, and an acute scarcity of longitudinal, multi-year field yield trials following elicitor withdrawal. Addressing these empirical bottlenecks through standardized multi-omics frameworks and rigorous life-cycle environmental assessments will be essential to transform empirical hardening practices into sustainable, genomics-informed biofactories for certified pre-basic seed potato production.

## Data Availability

No new data were created or analyzed in this study. Data sharing is not applicable to this article.

## Supplementary Materials

The following supporting information can be downloaded at: https://www.mdpi.com/article/10.3390/ijms27209288/s1.

## Abbreviations

The following abbreviations are used in this manuscript:

ABA	Abscisic acid
BAP	6-Benzylaminopurine
CAT	Catalase
CDPK	Calcium-dependent protein kinase
Dof	DNA-binding with one finger
DON	Deoxynivalenol
ET	Ethylene
GABA	γ-aminobutyric acid
IAA	Indole-3-acetic acid
IoT	Internet of Things
ISSR	Inter Simple Sequence Repeat
JA	Jasmonic acid
JmjC	Jumonji C domain-containing protein
LT50	Median lethal temperature
MDA	Malondialdehyde
MeJA	Methyl jasmonate
MS	Murashige and Skoog
MSAP	Methylation-sensitive amplification polymorphism
MVA	Mevalonate
NAA	1-Naphthaleneacetic acid
ncRNA	Non-coding RNA
PGPR	Plant-growth-promoting rhizobacteria
POD	Peroxidase
PRISMA	Preferred Reporting Items for Systematic Reviews and Meta-Analyses
RAPD	Random Amplified Polymorphic DNA
RLKs	Receptor-like kinases
ROS	Reactive oxygen species
RT-qPCR	Reverse transcription quantitative real-time PCR
SA	Salicylic acid
SCoT	Start Codon Targeted
SOD	Superoxide dismutase
TIBs	Temporary immersion bioreactors
UHPLC	Ultra-high-performance liquid chromatography
WGBS	Whole-genome bisulfite sequencing
