# Supplementary material for: Transcriptomic and Epigenetic Reprogramming in Solanum tuberosum L. Micropropagation: A Systematic Review of Elicitor-Driven Acclimatization and Scalable Biofactories
Source: Int J Mol Sci. 2026 Oct 19;27(20):9288. doi: 10.3390/ijms27209288 (PMC13704943; doi:10.3390/ijms27209288)
Supplement: Supplementary file 1 [file ijms-27-09288-s001.zip › Supplementary_File_S2_Table_S1_Excluded_Studies.pdf]

Supplementary Table S1. Detailed inventory of full-text records excluded during the eligibility assessment phase, with associated scientific rationales (n = 24), fulfilling PRISMA Item 16b requirements.

Reference (Author, Year)	Classification of Exclusion	Specific Technical Rationale
**I. Yield-centric or genetic focus lacking physiological/multi-omics profiling (n = 12)**		
Mali et al. (2024)	Genetic/Yield focus	Genome-wide identification of histone modification genes evaluated for heat stress response rather than *in vitro* acclimatization.
Kumar et al. (2010)	Genetic focus	Bt transgenic potato development evaluating cry1Ab promoter activity focused on pest resistance rather than physiological acclimatization.
Pervaiz et al. (2023)	Yield-centric focus	Microtuberization potential evaluated via jasmonic acid without subsequent *ex vitro* multi-omics profiling.
Adamo et al. (2024)	Agronomic focus	Evaluation of *Bacillus amyloliquefaciens* restricted to field yield variations across geographic regions.
Li et al. (2021)	Entomological focus	Analysis of steroidal glycoalkaloids focused on larval midgut detoxification in potato tuber moth (Phthorimaea operculella) rather than plantlet acclimatization.
Chen et al. (2015)	Genetic focus	Isolation and characterization of chalcone isomerase gene promoter restricted to tuber development.
Kaur et al. (2020)	Genetic focus	Genetic transformation and regeneration protocol in potato cv. Kufri Chipsona 1 lacking ex vitro physiological hardening data.
Fernández et al. (2015)	Genetic focus	Subtilisin-like protein characterization (StSBTc-3) focused on cell death mechanisms rather than *ex vitro* acclimatization.
Keller et al. (2014)	Conservation focus	Long-term cryopreservation protocols evaluated without active transcriptomic stress response datasets.
Thiruvengadam et al. (2025)	Yield-centric focus	Transcriptomic evaluation restricted to calcium signaling during the tuberization phase, lacking *ex vitro* transition data.
Nielsen et al. (2005)	Post-harvest focus	Global transcript profiling limited strictly to post-harvest tuber tissue without *in vitro* elicitation.
Krits et al. (2007)	Genetic focus	Analysis of steroidal glycoalkaloid levels and isoprenoid metabolic genes focused on generic tuber yield traits.

**II. Utilization of non-target biological models (non-Solanum tuberosum) (n = 8)**		
Luna-Luna et al. (2017)	Non-target species	Anatomy and morphology of acclimatized plants evaluated in *Agave potatorum* Zucc.
Burygin et al. (2019)	Non-target organism	Growth promotion and abiotic stress resistance evaluated primarily on *Ochrobactrum cytisi* interactions.
Chamkhi et al. (2021)	Non-target species	Microbial elicitor-induced secondary metabolites assessed globally in generic medicinal plants.
Tripathi et al. (2024)	Non-target species	Phytotoxicity and alleviation strategies of silver nanoparticles evaluated across generic agricultural models.
Iula et al. (2022)	Non-target species	Systemic acquired resistance and lipid remodeling under molecular elicitation evaluated strictly in tomato (Solanum lycopersicum).
Chávez et al. (2022)	Non-target species	Suspension cell culture and secondary metabolite elicitation evaluated in sweet potato (*Ipomoea batatas*) rather than *Solanum tuberosum*.
Goswami et al. (2020)	Non-target species	Plant growth-promoting rhizobacteria (PGPR) mechanisms reviewed generally across generic soil microbiomes.
Ghosh et al. (2020)	Non-target species	Molecular interaction and cellular defense mechanisms triggered by elicitors evaluated specifically in *Oryza sativa* (Rice).
**III. Redundant datasets or absence of discrete elicitation protocols (n = 4)**		
Yang et al. (2025)	Redundant dataset	Broad overview utilizing synthetic biology to accelerate plant-microbe interactions without primary experimental data.
Boubakri et al. (2020)	Redundant dataset	Book chapter summarizing previously published mechanisms on priming-mediated stress tolerance.
Pandey et al. (2022)	Redundant dataset	Book chapter detailing climate-resilient phytoremediation devoid of specific *in vitro* acclimatization metrics.
Padmanabhan et al. (2016)	Incomplete dataset	Encyclopedia entry providing generic health and carbohydrate composition data for potatoes without experimental methods.
