# Supplementary material for: Transcriptomic and Epigenetic Reprogramming in Solanum tuberosum L. Micropropagation: A Systematic Review of Elicitor-Driven Acclimatization and Scalable Biofactories
Source: Int J Mol Sci. 2026 Oct 19;27(20):9288. doi: 10.3390/ijms27209288 (PMC13704943; doi:10.3390/ijms27209288)
Supplement: Supplementary file 1 [file ijms-27-09288-s001.zip › Supplementary_File_S3_Table_S2_Risk_of_Bias.pdf]

Supplementary Table S2. Critical appraisal of methodological rigor and risk of bias across the included corpus (*n* = 58).

Assessment Framework: Custom evaluation framework optimized for in vitro and molecular assays. Risk Stratification: L = Low Probability, M = Moderate Probability, H = High Probability, U = Undetermined.

Reference ID	D1: Randomized Explant Allocation	D2: Microenvironmental Parameter Control	D3: Biological Replicate Adequacy	D4: Statistical Robustness	D5: Transparent Outcome Reporting	Overall Risk Profile
[16]–[25]	L	L	L	L	L	**LOW**
[26]	L	H	L	L	L	**ELEVATED***
[27]–[30]	L	L	L	L	L	**LOW**
[31]	L	H	L	H	L	**ELEVATED***
[32]–[34]	L	L	L	L	L	**LOW**
[35]	L	H	H	L	L	**ELEVATED***
[36]	L	L	L	L	L	**LOW**
[37]	H	L	L	H	L	**ELEVATED***
[38]–[56]	L	L	L	L	L	**LOW**
[57]	H	L	H	L	L	**ELEVATED***
[58]–[60]	L	L	L	L	L	**LOW**
[61]	L	H	L	L	L	**ELEVATED***
[62]–[68]	L	L	L	L	L	**LOW**
[69]	H	L	H	L	L	**ELEVATED***
[70]–[73]	L	L	L	L	L	**LOW**

**Notes on Diagnostic Domains:*

- **D1:** Were micropropagated explants subjected to robust randomization prior to targeted elicitor treatments?
- **D2:** Were *in vitro* and greenhouse microenvironments (e.g., photosynthetic photon flux density, VPD, thermal cycles) explicitly standardized and reported?

- **D3:** Was the sample size and biological replication statistically powered to detect transcriptomic or epigenetic variations reliably?
- **D4:** Were advanced statistical models (e.g., multifactorial ANOVA, FDR corrections for RNA-seq) appropriately deployed and exact P-values disclosed?
- **D5:** Did the authors present a comprehensive dataset, actively avoiding the selective reporting of favorable phenotypic outcomes?
